# Optimization of a nanotechnology based antimicrobial platform for food safety applications using Engineered Water Nanostructures (EWNS)

**DOI:** 10.1038/srep21073

**Published:** 2016-02-15

**Authors:** Georgios Pyrgiotakis, Pallavi Vedantam, Caroline Cirenza, James McDevitt, Mary Eleftheriadou, Stephen S. Leonard, Philip Demokritou

**Affiliations:** 1 Center for Nanotechnology and Nanotoxicology, Harvard School of Public Health, Harvard University, Boston, MA, USA; 2 European University Cyprus, The School of Sciences, Nicosia, Cyprus; 3 National Institute of Occupation Safety and Health, Morgantown, WV.

## Abstract

A chemical free, nanotechnology-based, antimicrobial platform using Engineered Water Nanostructures (EWNS) was recently developed. EWNS have high surface charge, are loaded with reactive oxygen species (ROS), and can interact-with, and inactivate an array of microorganisms, including foodborne pathogens. Here, it was demonstrated that their properties during synthesis can be fine tuned and optimized to further enhance their antimicrobial potential. A lab based EWNS platform was developed to enable fine-tuning of EWNS properties by modifying synthesis parameters. Characterization of EWNS properties (charge, size and ROS content) was performed using state-of-the art analytical methods. Further their microbial inactivation potential was evaluated with food related microorganisms such as *Escherichia coli, Salmonella enterica, Listeria innocua, Mycobacterium parafortuitum*, and S*accharomyces cerevisiae* inoculated onto the surface of organic grape tomatoes. The results presented here indicate that EWNS properties can be fine-tuned during synthesis resulting in a multifold increase of the inactivation efficacy. More specifically, the surface charge quadrupled and the ROS content increased. Microbial removal rates were microorganism dependent and ranged between 1.0 to 3.8 logs after 45 mins of exposure to an EWNS aerosol dose of 40,000 #/cm^3^.

Microbial contamination is a leading cause of foodborne illnesses, caused by the ingestion of pathogenic microorganisms or their toxins. Foodborne diseases cause approximately 76 million illnesses, 325,000 hospitalizations, and 5,000 deaths in the United States alone each year^1^. Furthermore, the United States Department of Agriculture (USDA) estimates that the increased consumption of fresh produce is responsible for 48% of all the reported food illnesses in the USA^2^. The cost of illnesses and deaths in the US caused by food borne pathogens is very high, estimated by the Center for Disease Control and Prevention (CDC) to exceed 15.6 billion USD annually^3^.

Currently chemical^4^, radiation^5^, and thermal^6^ antimicrobial interventions used for food safety assurance are primarily implemented at limited Critical Control Points (CCPs) (typically postharvest and/or during packaging) of the production chain, and not in a continuous manner, leaving fresh produce susceptible to cross contamination^7^. To more effectively address foodborne diseases and food spoilage, there is a need for antimicrobial interventions that have the potential to be applied throughout the “Farm-to-Fork” continuum, with low environmental impact and cost.

Recently a chemical free, nanotechnology based antimicrobial platform that can inactivate bacteria on surfaces and in air using Engineered Water Nanostructures (EWNS) was developed. EWNS are synthesized using two processes in parallel, namely, electrospraying and ionization of water (Fig. 1a). It was previously shown that EWNS possess a unique set of physical and biological properties^8^,^9^,^10^. EWNS have an average of 10 electrons per structure and an average nanoscale size of 25 nm (Fig. 1b,c)^8^,^9^,^10^. In addition, Electron Spin Resonance (ESR) showed that the EWNS contain a large number of reactive oxygen species (ROS), primarily hydroxyl (OH•) and superoxide (O_2_^−^) radicals (Fig. 1c)^8^. The EWNS remain airborne for a long time, potentially colliding with microorganisms suspended in air and present on surfaces, delivering their ROS payload and resulting in microbial inactivation (Fig. 1d). These earlier studies have also shown the potential of EWNS to interact and inactivate a variety of gram-negative and gram-positive bacteria of public health significance, including *Mycobacteria*, on surfaces and in air^8^,^9^. Transmission Electron Microscopy imaging showed that the inactivation is due to the destruction of the cell membrane. Further, acute inhalation studies showed that EWNS at high dose showed no lung injury or inflammation^8^.

Recently, the ability of the EWNS antimicrobial platform to inactivate food related microorganisms on the surface of fresh produce was also demonstrated. It was also shown that the surface electric charge of EWNS could be utilized, in combination with an electric field, to achieve targeted delivery. More importantly, promising preliminary results of approximately 1.4 log reduction on organic tomatoes in 90 minutes of exposure at approximately EWNS 50,000 #/cm^3^ was observed for a variety of food related microorganisms such as *E. coli* and *Listeria*^11^. Also, preliminary sensory evaluation tests showed no sensory effects compared to the control tomatoes. Although these initial inactivation results, even at the very low EWNS dose of 50,000 #/cc were promising for food safety applications, it was evident that higher inactivation potential would be more beneficial to further reduce the risk of infection and spoilage.

Here, we focus our investigation on developing an EWNS generation platform to enable fine-tuning of the synthesis parameters and optimizing the physicochemical properties of EWNS in order to increase their antimicrobial potential. More specifically, the optimization was focused on enhancing their surface charge (to enhance the targeted delivery) and ROS content (to enhance inactivation efficiency). The optimized physicochemical properties (size, charge, and ROS content) were characterized using state-of-the-art analytical methods and their inactivation potential was assessed using common food related microorganisms such as *E. coli*, *S. enterica*, *L. innocua*, *S. cerevisiae* and *M. parafortuitum*.

## Materials and Methods

### EWNS Synthesis

EWNS were synthesized by concurrently electrospraying and ionizing highly purified water (18 MΩ cm^−1^). Electrospraying^12^ is commonly used to aerosolize liquids and to synthesize polymeric and ceramic particles^13^ and fibers^14^ of controlled size.

**Figure 1:**
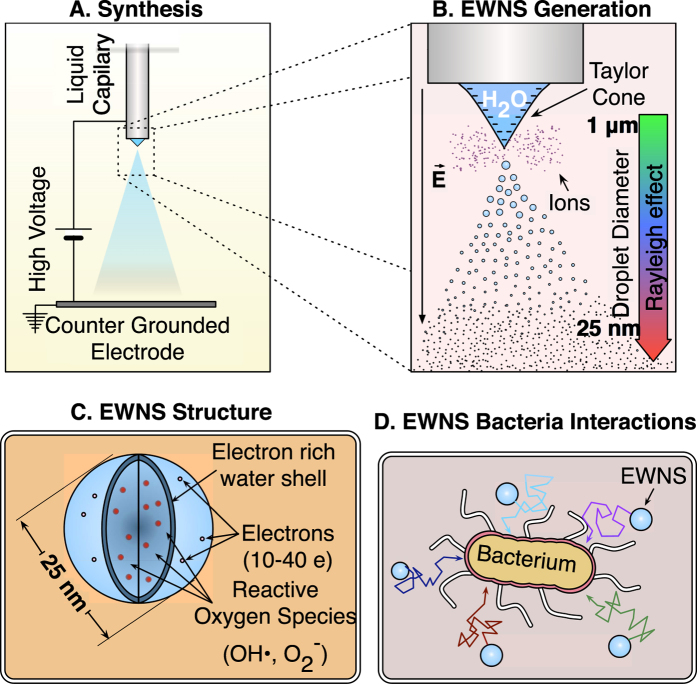
The EWNS synthesis principles. (**a**) Electrospray occurs when a high voltage is applied between a capillary containing the liquid and the counter electrode. (**b**) The application of the high voltage results into two distinct phenomena: (i) the electrospray of the water and (ii) generation of reactive oxygen species (ions) that are trapped in the EWNS. (**c**) The unique structure of EWNS. (**d**) EWNS due to their nanoscale nature are highly mobile and can interact with airborne pathogens.

As described in great detail in previous publications^8^,^9^,^10^,^11^, in a typical experiment, a high voltage is applied between a metal capillary and a grounded counter electrode. During the process, two distinct phenomena take place: i) the electrospraying and ii) the ionization of the water. The strong electric field between the two electrodes causes negative charges to accumulate on the surface of the condensed water, leading to the formation of the Taylor cone^15^. As a result, highly charged water droplets form and continue to break into smaller particles as the Rayleigh theory^16^. At the same time the high electric field causes some water molecules to split and can strip off electrons (ionization), resulting in a high number of reactive oxygen species (ROS)^17^. The concurrently generated ROS^18^ are encapsulated in the EWNS (Fig. 1c).

**Figure 2:**
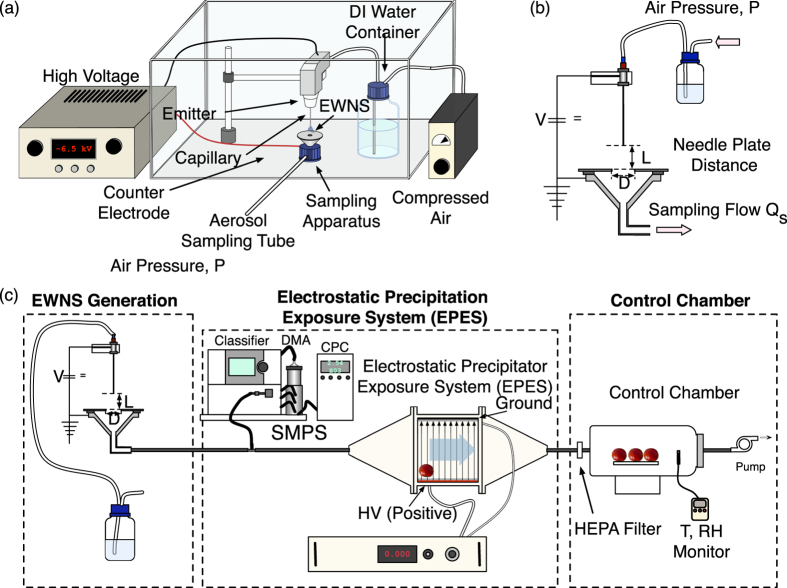
The experimental setup. (**a**) The Engineered Water Nano-Structures (EWNS) Generation System. (**b**) Cross section of the sampler and the electrospray device, showing the most important parameters. (**c**) The experimental setup that was used for the bacteria inactivation.

Figure 2a describes in detail the EWNS generation system that was developed and used in the synthesis of the EWNS for this study. The purified water, stored in tightly closed bottle, was fed through a Teflon tubing (ID 2 mm) to a 30G stainless steel needle (metal capillary). The flow of the water is controlled with the pressure of air inside the bottle as is shown in Fig. 2b. The needle is held on a Teflon cantilever that can be manually adjusted to a specific distance from the counter electrode. The counter electrode was a polished aluminum disk with an opening in the center to allow for sampling. Beneath the counter electrode there is an aluminum sampling funnel that is connected through a sampling port to the rest of the experimental apparatus (Fig. 2b). In order to avoid charge built-up that may impair the particle sampling, all the sampler components were electrically grounded.

### Synthesis Parameters

The EWNS generation system described above enables the modification of critical operational parameters to facilitate the fine-tuning of the EWNS properties. The applied voltage (V), the distance between the needle and counter electrode (L), and the flow of the water through the capillary (φ), were adjusted in order to fine tune the EWNS properties. The notation used to indicate the different combinations is [V (kV), L (cm)]. The water flow was adjusted to yield a stable Taylor cone for the particular [V, L] set. For the purpose of this investigation, the diameter of the counter electrode aperture (D) was kept at 0.5 inches (1.29 cm).

Due to the finite geometry and asymmetry, the electric field strength cannot be calculated from first principles. Instead the software QuickField™ (Svendborg, Denmark) was used to calculate the electric field^19^. The Electric field is not homogenous so as a reference value for the various configurations, the value of the electric field at the tip of the capillary was used.

During the investigation several combinations of the voltage and the distance between the needle and the counter electrode were evaluated in terms of Taylor cone formation, Taylor cone stability, EWNS production stability and reproducibility. The various combinations are summarized in Supplementary Table S1.

### EWNS Physicochemical characterization

#### Surface charge measurements of EWNS

The output of the EWNS generation system was connected directly to a Scanning Mobility Particle Sizer (SMPS, Model 3936, TSI, Shoreview, MN) to measure the particle number concentration, and in parallel to a Faraday Aerosol electrometer (TSI, Model 3068B, Shoreview, MN) used to measure the aerosol current, as described in our previous publication^9^. The SMPS and the Aerosol Electrometer were both sampling at 0.5 l/min flow rate (total sampling flow 1 l/min). The particle number concentration and the aerosol current were measured for the duration of 120s. The measurement was repeated 30 times. From the current measurement the total electric charge of the aerosol was calculated and the average EWNS electric charge was estimated for the given total number of EWNS particles sampled. The average EWNS charge 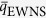 can be calculated with equation (1):


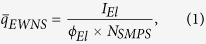


where *I*_*El*_ is the measured current, *N*_*SMPS*_ is the number concentration measured with the SMPS, and *φ*_*El*_ is the flow to the electrometer.

Since Relative Humidity (RH) can affect the surface electric charge, temperature and (RH) were maintained constant during the experiments, at 21 °C and 45%, respectively.

#### Size measurements of EWNS

An Atomic Force Microscope (AFM), Asylum MFP-3D (Asylum Research, Santa Barbara, CA) and the AC260T probes (Olympus, Tokyo, Japan) were used to measure the size and lifetime of EWNS. The AFM scan rate was 1 Hz and the scanned area 5 μm × 5 μm with 256 scan lines. All images were subjected to 1^st^ order image flattening with the Asylum Software (range of 100 nm and threshold of 100 pm for the mask).

The sampling funnel was removed and the mica surface was placed at a 2.0 cm distance from the counter electrode for an average time of 120s to avoid particle coalescence and the formation of irregular shaped droplets on the mica surface. The EWNS were directly sprayed on a freshly cleaved mica surface (Ted Pella, Redding, CA). The mica surface was imaged immediately after the spray using AFM. The contact angle of a freshly cleaved, unmodified, mica surface is close to 0^°^ so the EWNS were spread on the mica surface adopting a dome like shape^20^. The diameter (*a*) and the height (*h*) of the spread droplet were measured directly from the AFM topography and used to calculate the volume of the dome-like spread of EWNS with our previously validated methodology^8^. Assuming that airborne EWNS have the same volume, an equivalent diameter can be calculated with equation (2):


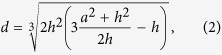


where *a* is the radius of the spread droplet and *h* the height.

#### ROS characterization of EWNS

Electron Spin Resonance (ESR) spin trapping was used to detect the presence of short-lived free radical intermediates in the EWNS based on our previously developed method. The aerosol was bubbled through a 650 μm Midget bubbler (Ace Glass, Vineland, NJ) containing a solution of 235 mM DEPMPO(5-(Diethoxyphosphoryl)-5-methyl-1-pyrroline-N-oxide), (Oxis International Inc. Portland, OR). All ESR measurements were conducted using a Bruker EMX spectrometer (Bruker Instruments Inc. Billerica, MA, USA) and a flat cell assembly. The Acquisit program (Bruker Instruments Inc. Billerica, MA, USA) was used for data acquisitions and analyses. The ROS characterization was performed only for the [−6.5 kV, 4.0 cm] set of operational conditions. The EWNS concentration was measured with the SMPS after considering the EWNS loses in the impinger.

#### Ozone monitor

The ozone levels were monitored with the 205 Dual Beam Ozone Monitor™ (2B Technologies, Boulder, Co)^8^,^9^,^10^.

#### Statistical Analysis

For all of the EWNS properties, the mean of the measurement was used as the measured value and the standard deviation was used as the error of the measurement. T-test was done to compare the values of the optimized EWNS properties to the respective values of the Baseline-EWNS.

### Electrostatic Precipitator Exposure System (EPES)

Figure 2c illustrates the previously developed and characterized “draw through” Electrostatic Precipitation Exposure System (EPES) which can be used for targeted delivery of EWNS on surfaces^11^. The EPES utilizes the electric charge of the EWNS and with the application of a strong electric field, it can directly “guide” them to the target surface. Details of the EPES system are presented in the recent publication by Pyrgiotakis *et al.*^11^. In summary, the EPES consists of a 3D printed PVC chamber, which has tapered ends and contains two parallel stainless steel metal plates (Stainless Steel 304, Mirror Finish) in the center, placed at 15.24 cm apart. The plates are connected to an external high voltage source (Bertran 205B-10R, Spellman, Hauppauge, NY), with the bottom plate always connected to positive voltage and the top plate is always connected to ground (floating ground). The walls of the chamber were coated with aluminum foil that was electrically grounded to prevent particle loses. The chamber has an airtight front-loading door that allows the test surfaces to be placed on a plastic rack that keeps them elevated from the lower metal plate in order to avoid interference from the high voltage.

The deposition efficiency of the EWNS in the EPES was calculated according to the previously developed protocols that are described in detail in the Supplementary Figure S1^11^.

As control chamber, a second flow through cylindrical chamber was connected in series to the EPES system, utilizing a HEPA filter in-between, to remove the EWNS. The EWNS aerosol is drawn through both in-line chambers with a pump as is illustrated in Fig. 2c. The filter between the control chamber and the EPES removes all the remaining EWNS resulting in identical Temperature (T), Relative Humidity (RH) and Ozone levels.

### Bacterial Inactivation Protocols

Important food related microorganisms found to contaminate fresh produce such as *Escherichia coli* (ATCC #27325), a fecal indicator, *Salmonella enterica* (ATCC #53647), a foodborne pathogen, *Listeria innocua* (ATCC #33090), a surrogate to pathogenic *Listeria monocytogenes*, *Saccharomyces cerevisiae* (ATCC #4098), a surrogate to spoilage yeast and *Mycobacterium parafortuitum* (ATCC #19686) a more resilient bacterium to inactivate, were procured from ATCC (Manassas, VA).

Random boxes of organic grape tomatoes were bought from the local market and refrigerated at 4 °C until use (maximum of 3 days). The experimental tomatoes were selected to be of the same size, approximately 1/2 inch in diameter.

The culture, inoculation, exposure, and colony enumeration protocols have been described in detail in our previous publication and are explained in detail in the supplemental data^11^. The EWNS efficacy was evaluated by exposing inoculated tomatoes to 40,000 #/cc for 45 mins. In brief, three tomatoes were used to assess the surviving microorganisms, at the time point t = 0 mins. Three tomatoes were placed in the EPES and exposed to EWNS at 40,000 #/cc (EWNS exposed tomatoes) and three more in the control chamber (control tomatoes). There was no additional treatment for either group of tomatoes. The EWNS–exposed and control tomatoes were removed after 45 mins to assess the effect of the EWNS.

Each experiment was done in triplicate. The data analysis was done according to the protocol described in the supplemental data.

### Transmission Electron Microscopy (TEM) Imaging

The EWNS exposed (45 mins at EWNS aerosol concentration of 40,000 #/cm^3^) and the unexposed bacterial samples of *E. coli*, *S. enterica* and *L. innocua* were pelleted to assess the inactivation mechanism. The pellets were fixed for 2 h at RT in 2.5% glutaraldehyde, 1.25% paraformaldehyde, and 0.03% picric acid in 0.1 M sodium cacodylate buffer (pH7.4) fixative. After washing they were post fixed with 1% osmiumtetroxide (OsO_4_)/1.5% potassiumferrocyanide (KFeCN_6_) for 2 h, washed in water 3 times and incubated in 1% uranyl acetate for 1 h followed by two washes in water and subsequent dehydration for 10 minutes each in 50%, 70%, 90%, 100% alcohol. The samples were then put in propyleneoxide for 1 h and infiltrated in a 1:1 mixture of propyleneoxide and TAAP Epon (Marivac Canada Inc. St. Laurent, CA). The samples were embedded in TAAB Epon and polymerized at 60 °C for 48 h. The solidified pelleted resins were sliced and imaged by TEM using JEOL 1200EX (JEOL, Tokyo, Japan), a conventional transmission electron microscope equipped with an AMT 2k CCD camera (Advanced Microscopy Techniques, Corp., Woburn, MA, USA).

### Statistical Analysis of microbiological data

All the experiments were performed in triplicates. For each time point the bacteria rinsate solution was plated in triplicate resulting in a total of nine data-points for each point the average of which was used as the bacterial concentration of that particular microorganism. The standard deviation was used as the measurement error. All points were accounted for.

The Log Reductions of bacterial concentrations compared to t = 0 mins were computed according to:


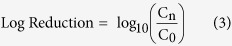


where C_0_ is the bacteria concentration of the control coupon at time 0 (i.e. after surface was dried, but before placed into a chamber) and C_n_ is the bacteria concentration of the surface after n minutes of exposure.

To account for the natural decay of the bacteria during the 45 min exposure, the Log-Reduction was also calculated as compared to the control at 45 mins as follows:


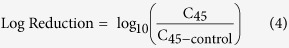


where C_n_ is the bacteria concentration of the control coupon at time n while C_n-Control_ is the concentration of the control bacteria at time n. The data are presented in terms of log reduction compared to the control (no-EWNS exposure).

## Results

### Physicochemical characterization of EWNS

**Figure 3:**
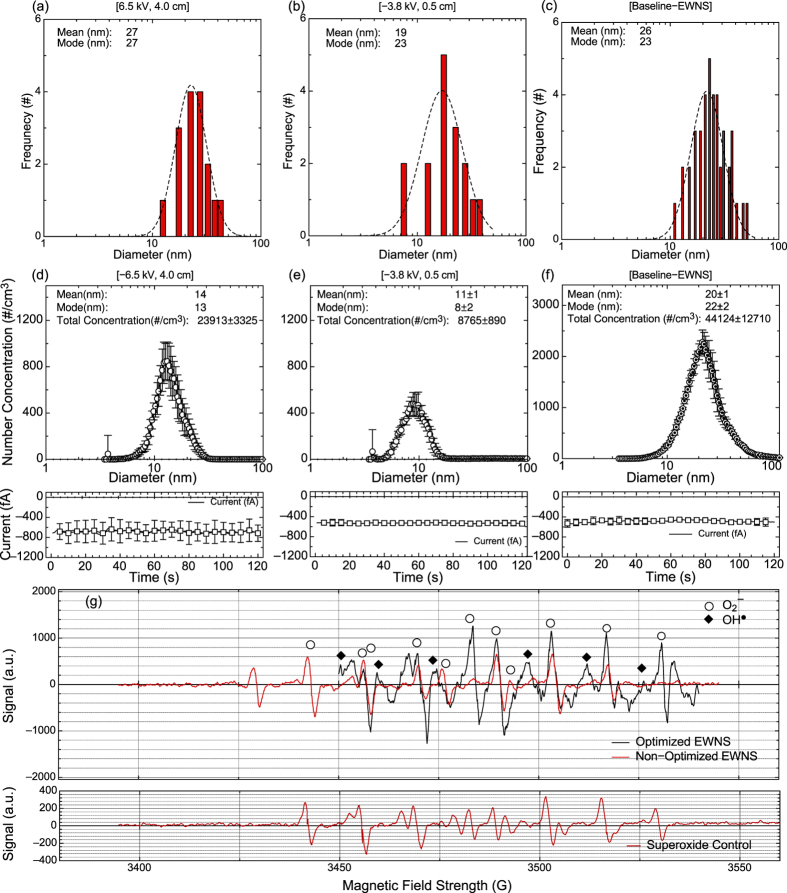
The physicochemical characterization of the EWNS. (**a–c**) The size distribution as measured with the AFM. (**d–f**) The surface charge characterization. (**g**) The ROS characterization with the EPR.

During the investigation several combinations of the voltage and the distance between the needle and the counter electrode were evaluated in terms of Taylor cone formation, Taylor cone stability, EWNS production stability and reproducibility. The various combinations are summarized in Supplementary Table S1. The two cases that displayed stable and reproducible properties (Taylor cone, EWNS production and stability over time) were selected for the full investigation. Figure 3 shows in detail the results for the charge, size and ROS content for these two cases. The results are also summarized in Table 1. As a reference, both Fig. 3 and Table 1 include the properties of the previously synthesized, non-optimized EWNS^8^,^9^,^10^,^11^, (Baseline-EWNS). The statistical significance calculation with the two tailed t-test is reposted in the Supplementary Table S2. Further, the supplemental data include an investigation of the effects of counter electrode sampling hole diameter (D) and distance between the grounded electrode and the tip of the needle (L) (Supplementary Figure S2 and S3).

It is also important to note that for all the above mentioned conditions the measured ionization electric current was between of 2–6 μΑ and the voltage was between −3.8 and −6.5 kV, resulting in energy consumption less than 50 mW for this single needle EWNS generation module. Although the EWNS were synthesized with the application of high voltage the ozone levels were very low and never exceeded the 60 ppb levels.

#### Electric Field

Supplementary Figure S4 show the simulated electric field for the case of the [−6.5 kV, 4.0 cm], and [−3.8 kV, 0.5 cm] scenarios respectively. The field was calculated to be 2 × 10^5^ V/m, and 4.7 × 10^5^ V/m for the [−6.5 kV, 4.0 cm] and [−3.8 kV, 0.5 cm] scenarios respectively. This is expected since the voltage distance ratio is significantly higher for the second case.

#### EWNS Size

Figure 3a,b show the EWNS diameter as it is measured with the AFM^8^. The average EWNS diameter was calculated to 27 nm and 19 nm respectively for the [−6.5 kV, 4.0 cm] and [−3.8 kV, 0.5 cm] scenarios. The geometric standard deviation of the distribution was 1.41 and 1.45 respectively for the [−6.5 kV, 4.0 cm] and [−3.8 kV, 0.5 cm] scenarios, which is indicative of a narrow size distribution. Both the average size and the geometric standard deviation are very close to the Baseline-EWNS, 25 nm and 1.41 respectively. Figure 3c shows the size distribution measured with the same methodology under the same conditions for the Baseline-EWNS.

#### EWNS electric charge

Figure 3d,e show the results of the charge characterization. The data represent the average measurement of 30 number Concentration (#/cm^3^) and Current (I) concurrent measurements. The analysis showed that the average charge per EWNS is 22 ± 6 e^−^ and 44 ± 6 e^−^ respectively for the [−6.5 kV, 4.0 cm] and [−3.8 kV, 0.5 cm], respectively. As compared to the Baseline-EWNS (10 ± 2 e^−^) they have significantly higher surface charge that is two times for the [−6.5 kV, 4.0 cm] scenario and four times higher for the [−3.8 kV, 0.5 cm] scenario. Figure 3f shows the electric charge data for the Baseline-EWNS.

#### EWNS number concentration

It is evident from the EWNS number concentration graphs (Supplementary Figure S5 and S6) that the particle number for the [−6.5 kV, 4.0 cm] scenario is significantly higher compared to the [−3.8 kV, 0.5 cm] scenario. It is also worth noting that the EWNS number concentration was monitored for up to 4 hours (Supplementary Figure S5 and S6) where the EWNS generation stability showed similar particle number concentration levels for both scenarios.

#### ROS Investigation

Figure 3g shows the ESR spectrum, after subtraction of control (background) of the optimized EWNS for the [−6.5 kV, 4.0 cm]. The ROS spectrum was also compared to that of Baseline-EWNS scenario from previously published work. The number of the EWNS reacted with the spin trap was calculated, at 7.5 × 10^4^ EWNS/s which is similar to the one previously reported for the Baseline-EWNS^8^. The ESR spectrum clearly indicates the presence of two ROS species, with O_2_^−^ being the dominant species, and OH^•^ present in smaller amounts. Further the direct comparison of the peak intensity indicates that the optimized EWNS have significantly higher ROS content compared to the Baseline-EWNS.

#### EWNS deposition in EPES

**Figure 4:**
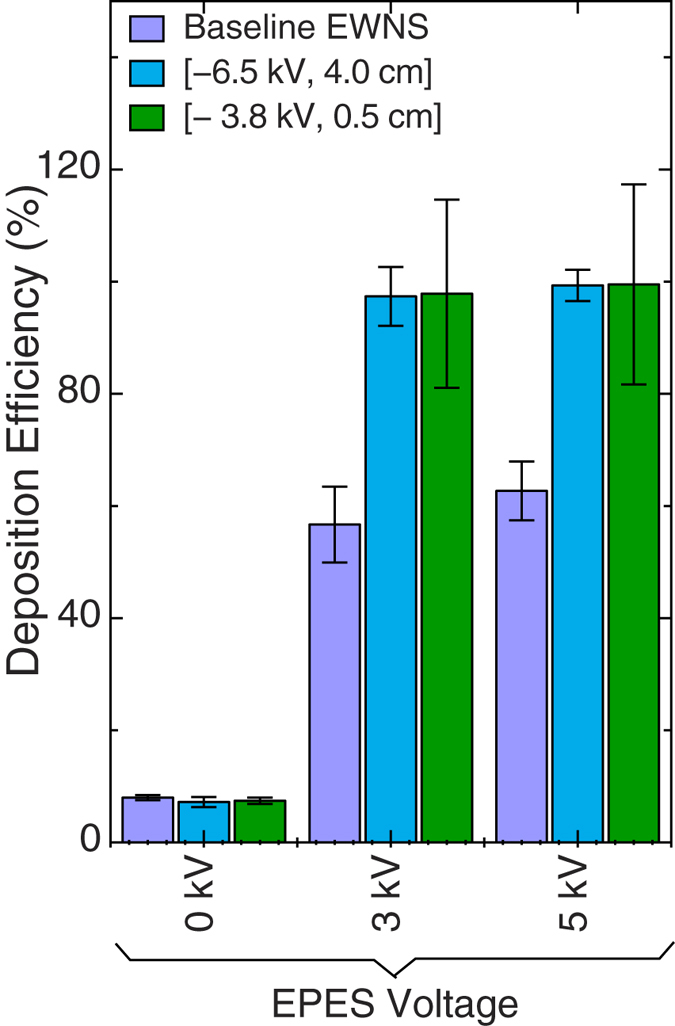
The deposition of the EWNS in the EPES.

Figure 4 represents the EWNS deposition efficiency in the EPES. The data are also summarized in Table I and compared with the Baseline-EWNS data. The deposition even for the low voltage of 3.0 kV is reaching nearly 100% deposition for both cases of the EWNS. Generally, the 3.0 kV is enough to reach 100% deposition regardless of the variations on the surface charge. Under the same conditions the Baseline-EWNS had only 56% deposition efficiency due to their lower electric charge (10 electrons per EWNS on average).

### Microbial Inactivation

**Figure 5:**
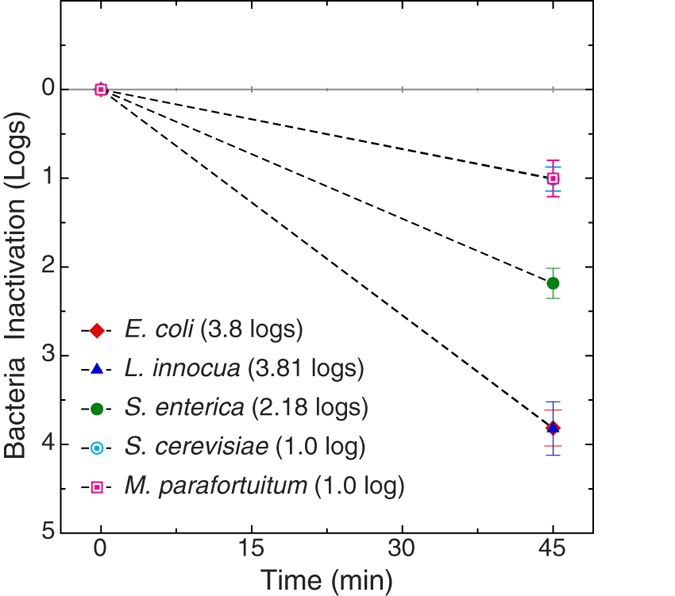
Inactivation of bacteria on tomato surface in EPES with the optimized EWNS. The data presented are normalized to the control.

The magnitude of inactivation of the inoculated, on the tomato surface, microorganisms following exposure to approximately 40,000 #/cm^3^ EWNS for 45 min for the [−6.5 kV, 4.0 cm] optimum scenario, are summarized in Fig. 5 and Table 2. The inoculated *E. coli* and *L. innocua* showed a significant 3.8 log reduction in 45 min of exposure. At the same conditions, *S. enterica* exhibited a lower log reduction of 2.2 logs, while both *S. cerevisiae* and *M. parafortuitum* showed a 1.0 log reduction.

### Electron Microscopy

**Figure 6:**
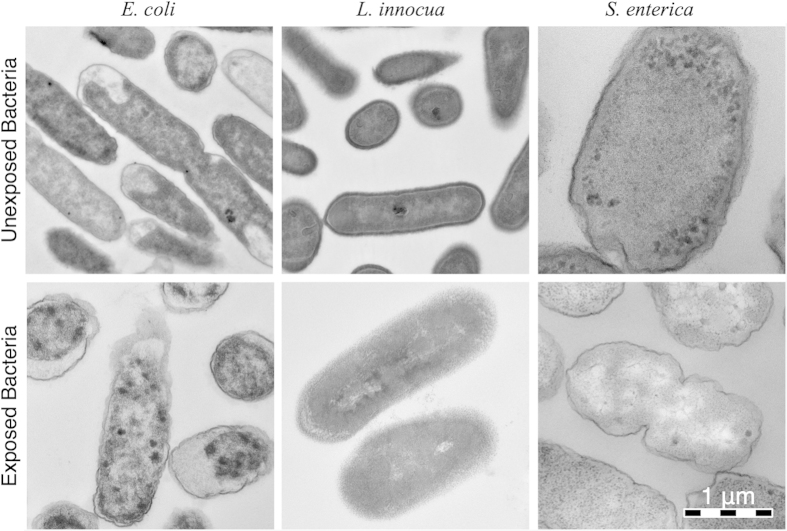
The electron microscopy imaging of the control and exposed bacteria revealing the damage to the membrane.

Electron micrographs (Fig. 6) depict the physical changes induced by the EWNS to the cells of *E. coli*, *S. enterica* and *L. innocua*, causing inactivation. The control bacteria showed an intact cell membrane, while the exposed bacteria have a damaged outer membrane.

## Discussion

The data regarding the physicochemical characterization of the optimized EWNS, collectively, show that the EWNS properties (surface charge and ROS content) have been enhanced significantly compared to the previously reported EWNS baseline data^8^,^9^,^10^,^11^. On the other hand, their size still remains in the nano regime, very similar to the previously reported results, allowing them to remain airborne for a long time. The observed polydispersity can be explained from the variation of the surface charge^21^, which dictates the size, the randomness of the Rayleigh effect and potential coalescences of the EWNS. However, as it was described in detail by Nielsen *et al.*^22^ the high surface charge reduces the evaporation by effectively increasing the surface energy/tension of the water droplets. This theory has been validated experimentally for micro-sized droplets^22^ and for the EWNS in our previous publication^8^. The overtime loss of charges might as well affect the size and contribute to the observed size distribution.

Further the electrical charge was in the order of 22–44 e^−^ per structure, depending on the scenario, significantly higher compared to the Baseline-EWNS, which had an average charge of 10 ± 2 electrons per structure. It should, however, be noted that this represents the average charge of the EWNS. Seto *et al.* have shown that the charge is not uniform and follows a log-normal distribution^21^. Compared to our previous work, the doubling surface charge doubles the deposition efficiency in the EPES system, reaching an almost 100%^11^.

The optimization of the synthesis parameters, also affected the ROS content. As it can be seen from the EPR spectra comparison (optimized EWNS vs. previously reported Baseline-EWNS), both EWNS aerosols have similar compositions, comprising of OH• and O_2_^−^ radicals. Although the exact concentration cannot be determined directly from these results, the peak intensity is related to the ROS concentration. The peak height of the optimized EWNS is higher, which implies that the optimized EWNS have higher ROS content. Quantification of these differences and detailed concentration calculations requires further investigation that was beyond the scope of this paper. Although the direct measurement of the lifetime of the ROS within the EWNS remains technically a challenge, the presence of ROS in EWNS after their synthesis was indirectly confirmed in a previous study, where the inactivation of microorganisms occurred up to 38 minutes after their production^8^.

Ozone levels were below 60 ppb, which is expected due to the lower voltage applied in the current generation system. This is an important improvement, eliminating the need for ozone scrubbers^8^, as these levels are below the EPA allowed (72 ppb for a maximum of 8 hours)^23^.

As expected the micro-organisms displayed different response to the EWNS exposure^24^, which is in agreement with literature showing that there is a significant variation among different microbial species to physical and chemical inactivation methods^11^. Bacterial spores show the highest resistance, followed by mycobacteria^25^. It is well known that mycobacteria have a protective waxy coating^26^, which makes them hard to inactivate^27^,^28^ and is responsible for the resilience they display to chemical, radiation treatments^29^,^30^,^31^. In regards to yeast, similar inactivation studies using treatments involving free radicals and H_2_O_2_ have also found yeasts to be more resistant to inactivation compared to vegetative bacteria attributing this to structural differences and more specifically to cell wall compositional differences^32^.

In an earlier publication regarding microbial inactivation on fresh produce surfaces using the Baseline-EWNS, we had observed lower inactivations to EWNS exposure. More specifically, *E. coli* was reduced by 0.7 logs following a similar 45 mins exposure to 50,000 #/cm^3^ of EWNS, while *L. innocua* was reduced by 0.2 logs after 45 min exposure to 23,000 #/cm^3^ of EWNS^11^. It is clearly evident that the optimized EWNS in this study were able to confer significantly higher log reductions in comparison to the Baseline-EWNS. The five-fold increased inactivation efficacy observed in this study can be attributed to the optimization of the EWNS properties. The increased electric charge for the optimized EWNS (22 e^−^/particle) compared to the Baseline-EWNS (10 e^−^/EWNS) has an effect on the deposition efficiency in the EPES system (the deposition efficiency increased from 56% to 99%). That alone may explain some of the increased inactivation potential for the optimized EWNS used in this study. However, even when the inactivation potential was normalized per particle delivered (which are 2 × 10^7^ #/min for optimized EWNS and 5 × 10^7^ #/min for the Baseline-EWNS) there is a significant difference between the two EWNS particle systems. For *E. coli*, the optimized EWNS resulted into 4.2 × 10^−9^ logs/EWNS, while the previously reported baseline-EWNS data show 0.3 × 10^−9^ logs/EWNS. It is evident that there is approximately 13 times higher inactivation potential from the optimized EWNS, aside from the higher deposition efficiency, indicative of a higher ROS content, as shown in the property characterization section above. Similar enhancement was also observed for the inactivation of *Listeria* with 40 times increase in the inactivation potential (4.2 × 10^−9^ logs/EWNS for the optimized EWNS vs. 0.1 × 10^−9^ logs/EWNS for the Baseline-EWNS)^11^. It is clear therefore, that the increased ROS concentration of the optimized EWNS have a significant contribution to the higher observed inactivation rates as compared to the previously reported Baseline-EWNS.

It has been shown that high levels of ROS can cause stress conditions in gram-negative microorganisms by augmenting irreversible damage to cellular components due to reduction of cell stability and modification of proteins^33^. All the control cells appeared normal and had an intact internal structure and cell membrane, whereas the cells exposed to EWNS appeared to have their cell membrane damaged, in agreement with the previously published data. The three pathogens: *E. coli*, *S. enterica* and *L. innocua* exposed to EWNS display cracked and ruptured membranes as compared to the control cells that had their membranes intact, clearly defined periplasmic space, and intercellular structure. In a previous publication the destruction of the membrane lipid due to the presence of ROS was assessed with a lipid peroxidation assays (LPO), identifying the ROS as the primary mechanism of inactivation^10^.

Typical chemical treatment of fresh produce such as peroxyacetic acid (PAA) and chlorine wash can inactive *E. coli O157:H7*, *Listeria* and *Salmonella spp*. by achieving 1–3 logs removal within 3 minutes^4^. In particular chlorine is considered as the gold standard for cleaning fresh fruits, with exceptional results^34^. However, due to its nature it can only be applied at certain points in the farm-to-fork continuum, usually at post harvest/packaging. Further although Chlorine is permitted to be used for the disinfection of organic produce^35^, EPA sets the limit of the residual chlorine to 4 mg/l (drinking water limit)^36^, constraining the usage to dilute chlorine solutions that are essentially ineffective^37^.

EWNS on the other hand, can provide an alternative, stand alone or complimentary disinfection intervention strategy that can be advantageous to the food industry for many reasons. EWNS can be applied throughout the food production chain, including transportation, storage and marketplace displays, points that can introduce microbial contaminants and compromise prior attempts to reduce the presence of such contaminants^38^. In addition, EWNS can be applied as a continuous disinfection strategy i.e. marketplace display or warehouse storage. Further since there are no chemical residues left behind, it can be a suitable technology for organic produce. Further, the technology consumes very little energy, less than 50 mW for a single needle generation module. This is true even for up-scaling the technology where even 100 needles can consume less than 5 W (typical USB power). It should be noted, however, that the EWNS can only be used to inactivate surface microorganisms^7^,^39^. According to the CDC, these surface bacteria are collectively responsible for a large number of all food related diseases^40^. In particular *E. coli* and *listeria* have caused the two worst outbreaks of the last decade in Europe^41^ and USA^42^ respectively.

Future studies will focus on the development of a high throughput EWNS generation system using a multi-needle approach to increase the EWNS aerosol concentration output and achieve even higher inactivation in shorter exposure time.

In conclusion, the optimized EWNS properties resulted in higher ROS content and deposition efficiency and in higher microorganism inactivation potential as compared to the previously published Baseline-EWNS. Our current work focuses on inactivation of natural micro-flora present on the surface of fresh produce, both conventionally and organically grown. Given the fact that this technology consumes very little energy, utilizes just water, and leaves no chemical residue, it can become a promising solution in the battle against food borne infections.

## Additional Information

**How to cite this article**: Pyrgiotakis, G. *et al.* Optimization of a nanotechnology based antimicrobial platform for food safety applications using Engineered Water Nanostructures (EWNS). *Sci. Rep.* **6**, 21073; doi: 10.1038/srep21073 (2016).

## Supplementary Material

Supplementary Information

## Figures and Tables

**Table 1:** The operational parameters and the main physicochemical properties of the optimized EWNS and comparison with the previously reported work (baseline).

Electrospray Conditions		Electrospray Module Operational Parameters				EWNS Physicochemical characterization			Deposition in EPES	
Name	Reference	V (kV)	L (cm)	D (cm)	*φ* (*μ*l/min)	Diameter‡ (nm)	Charge (e^−^)	ROS	3 kV 0.5 l/min	5 kV 0.5 l/min
Baseline†	[−5.0 kV, 0.5 cm]	−5.0	0.5	0.40	N/A	26 ± 9	10 ± 2	OH•, O_2_^−^	52.2 ± 5.8	62.7 ± 5.2
Condition I	[−6.5 kV, 4.0 cm]	−6.5	4.0	1.29	1.2	27 ± 10*	22 ± 6*	OH•, O_2_^−^	97.4 ± 5.3*	99.3 ± 2.8*
Condition II	[−3.8 kV, 0.5 cm]	−3.8	0.5	1.29	0.9	19 ± 7*	44 ± 6*	N/I	97.8 ± 6.8*	99.5 ± 7.8*

Notation used at the table (also described in Fig. 2).

V: Applied Voltage.

L: Distance between counter electrode and needle.

D: Counter Electrode sampling diameter.

φ: Flow of the water.

^*^Statistically significant difference compared to the baseline (P < 0.01).

^†^Different electrode geometry.

^‡^Arithmetic Standard Deviation of the distribution. N/I Not investigated.

**Table 2:** Summary of Inactivation results.

Pathogen	Logs Inactivated after exposure at 40,000 #/cm^3^ for 45 mins	
	Logs Inactivated Compared to time 0 min	Logs Inactivated Compared to Control at 45 min
*E. coli*	4.8 ± 0.2	3.8 ± 0.2
*L. innocua*	5.5 ± 0.1	3.8 ± 0.3
*S. enterica*	3.6 ± 0.1	2.2 ± 0.2
*S. cerevisiae*	3.1 ± 0.4	1.0 ± 0.1
*M. parafortuitum*	3.6 ± 0.4	1.0 ± 0.3
