# Supplementary material for: Optimization of a nanotechnology based antimicrobial platform for food safety applications using Engineered Water Nanostructures (EWNS)
Source: Sci Rep. 2016 Feb 15;6:21073. doi: 10.1038/srep21073 (PMC4753486; doi:10.1038/srep21073)
Supplement: Supplementary Information [file srep21073-s1.docx]

**Optimization of a nanotechnology based antimicrobial platform for food safety applications using Engineered Water Nanostructures (EWNS)**

**(Supplemental data)**

Georgios Pyrgiotakis, Pallavi Vedantam, Caroline Cirenza, James McDevitt, Mary Eleftheriadou, Steven Leonard, Phillip Demokritou

**Characterization of the EWNS deposition efficiency of the EPES system**

The complete evaluation of the EWNS deposition efficiency as a function of the flow through chamber and the applied voltage has been reported elsewhere.^1^ Here the same protocols were followed to determine the deposition efficiency of the optimized EWNS in the EPES. The EWNS concentration was measured upstream (before the EPES, C_B_) and downstream (after the EPES, C_A_,) (Figure S1) of the electrostatic precipitation chamber using the SMPS (TSI, Shoreview, MN). The deposition efficiency (α) of the aerosol due to the Electric Field was calculated as follows:

| $\text{α=}\frac{\text{C}_{\text{B}}\text{-}\text{C}_{\text{A}}}{\text{C}_{\text{B}}}$ | (1) |
| --- | --- |

**Bacterial culture, inoculation of the tomatoes, exposure and colony enumeration protocols**

***Preparation of the inoculum***

*E. coli*, *S. enterica*, and *L. innocua* were streaked on to a Tryptic Soy Agar plate (TSA) (VWR, USA) and incubated at 37°C for 24 h. *M. parafortuitum* was streaked on TSA and incubated at 37°C for 4-6 days. *S. cerevisiae* was streaked on to Sabouraud dextrose agar (SDA) (VWR, USA) at 30°C for 48 h. A single isolated colony of each microbial strain was inoculated into sterile 20 ml Tryptic Soy broth (TSB) (VWR, USA) and incubated overnight in a shaker incubator at 37°C(*S. cerevisiae* at30°C) . The resulting culture was centrifuged at 3000 x g for 30 minutes. The supernatant was discarded and washed with sterile DI water (20 ml). A total of three washes were performed and the pellet was resuspended in 5 ml of sterile DI water. DI water was selected over PBS since the PBS leaves behind salt residue when drying, that can coat the bacteria and interfere with the experiments. The final concentration of inoculum was adjusted to 1 x 10^8^ CFU/ml for all cultures.

***Inoculation of tomato surface***

The tomatoes were washed in a diluted soap solution and rinsed three times in sterile distilled water and dried thoroughly before inoculation with culture. Control experiments (data not shown) confirmed that this protocol removes all pathogens. Distilled water was used to re-suspend the pellet instead of phosphate buffered saline as the sodium chloride can create a hypertonic condition upon drying and also may interfere with nanoparticle exposure to the bacterial inoculation on tomato surface. A total of 10 µl of culture was spot inoculated on each grape tomato in increments of 1 µl to ensure a thin layer of microbial culture, prevent the overlapping of cells and facilitate increased interaction with the EWNS. They were then left to air dry.

***Protocol for bacterial inactivation***

The complete experimental setup is shown in figure 2c. The detailed protocol of the bacterial inactivation experiment has been described in detail elsewhere.^1^ In brief, three tomatoes were set aside for time zero, three were placed in the EPES chamber and were exposed to the EWNS. The inoculated tomatoes were placed on a plastic rack at the edge of the bottom plate towards the EWNS aerosol inlet. The exposure was done for 40,000 #/cm^3^. Three more inoculated tomatoes were placed in the control chamber for 45 minutes.

After each exposure, the tomatoes were removed from each chamber. Each tomato was placed in a centrifuge tube containing 20 ml sterile phosphate buffered saline (PBS), vigorously vortexed for two minutes to recover the bacteria from the tomato surface and centrifuged at 3000 x g for 30 minutes to concentrate the bacteria. The supernatant were discarded and pellet was resuspended in sterile 200 µl PBS. This solution was used to streak plates for the CFU counting.

Spread plating was done for the bacterial rinsate in triplicates and the CFU/ml was calculated. As growth media SDA was used for *S. cerevisiae* and was incubated at 30°C for 48 h. TSA was used for all other pathogens for spread plating. *E. coli*, *S. enterica* and *L.innocua* were incubated at 37°C for 24 h. *M. parafortuitum* was incubated at 37°C for 4-6 days. Relative humidity of 25% was maintained for all the bacteria (except *S. eneterica* which was adjusted to 40%) to maintain good growth and viability.

**Table S1:** The combinations that were used for the optimization of the EWNS production. The data were obtained with the 1.27 cm aperture diameter of the counter electrode.

| **Distance**  **[cm]** | **Voltage Range***  **[kV]** | **Taylor Cone**  **Formation Volt.** | **Cone**  **Stability** | **Cone**  **Reproducibility** | **Average EWNS Conc. [#/cm^3^]** |
| --- | --- | --- | --- | --- | --- |
| 0.5 cm | 0.00 – 4.00 | 3.8 kV | Yes | Yes | 28,000 |
| 1.0 cm | 0.00 – 5.00 | - | - | - | N/A |
| 1.5 cm | 0.00 – 7.00 | 5.3 kV | No | No | N/A |
| 2.0 cm | 0.00 – 8.00 | 6.0 kV | Yes | Yes | 22,000 |
| 4.0 cm | 0.00 – 10.00 | 6.5 kV | Yes | Yes | 40,000 |

*Over the upper limit of the voltage the needle vibrates or there is arching.

**Table S2:** The t-test analysis of the properties of the optimized EWNS as compared to the Baseline-EWNS.

|  | **Baseline** | **Optimized EWNS** | | | |
| --- | --- | --- | --- | --- | --- |
|  |  | [-6.5 kV, 4.0 cm] | | [-3.8 kV, 0.5 cm] | |
|  | Mean ± SD | Mean ± SD | P – Value* | Mean ± SD | P – Value* |
| Diameter (nm) | 26±9 | 27±10 | 0.8168 | 19±7 | 0.0680 |
| Charge (e^-^) | 10±2 | 22±6 | <0.0001 | 44±6 | <0.0001 |
| Deposition EPES – 3kV (%) | 52.2±5.8 | 97.4±5.3 | <0.0001 | 99.3±2.8 | <0.0001 |
| Deposition EPES – 5kV (%) | 62.7±5.2 | 97.8±6.8 | <0.0001 | 99.5±7.8 | <0.0001 |
| The P – Value is calculated to the respective baseline value | | | | | |


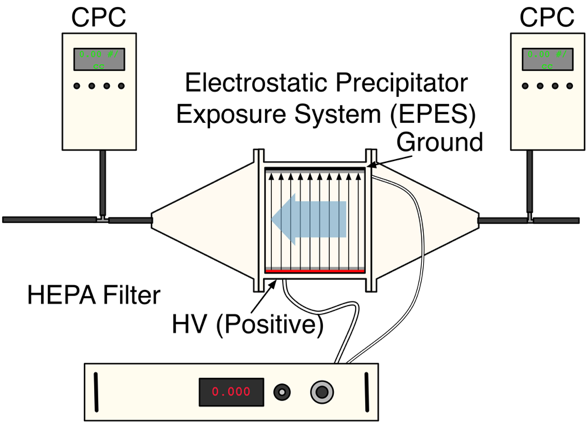


Before

After

**Figure S1:** Deposition efficiency characterization setup.


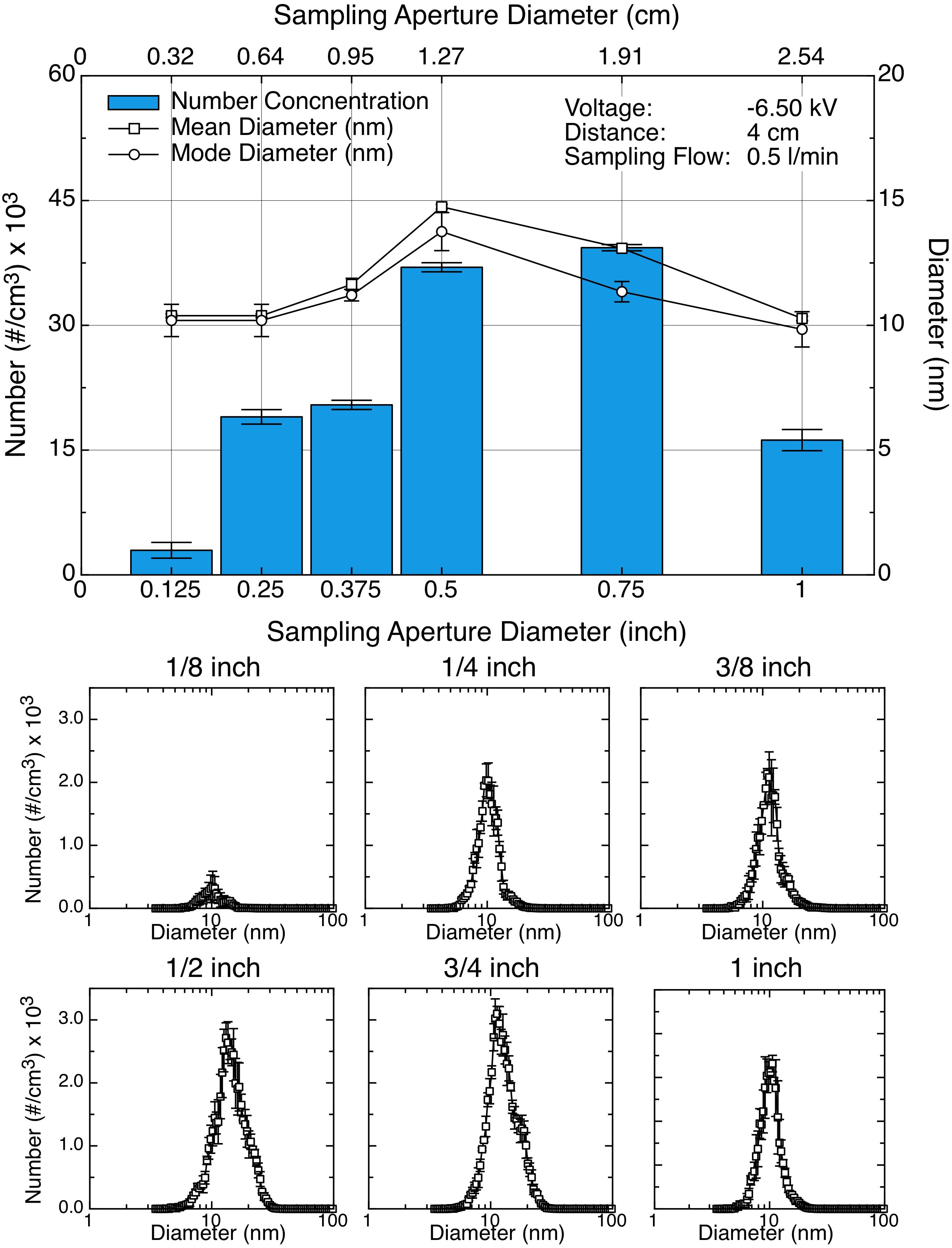


**Figure S2:** Effect of the counter electrode-sampling hole on the number of produced EWNS on the [-6.5 kV, 4 cm] scenario. The diameter is indicative, as the SMPS is not a valid tool for the measurement.


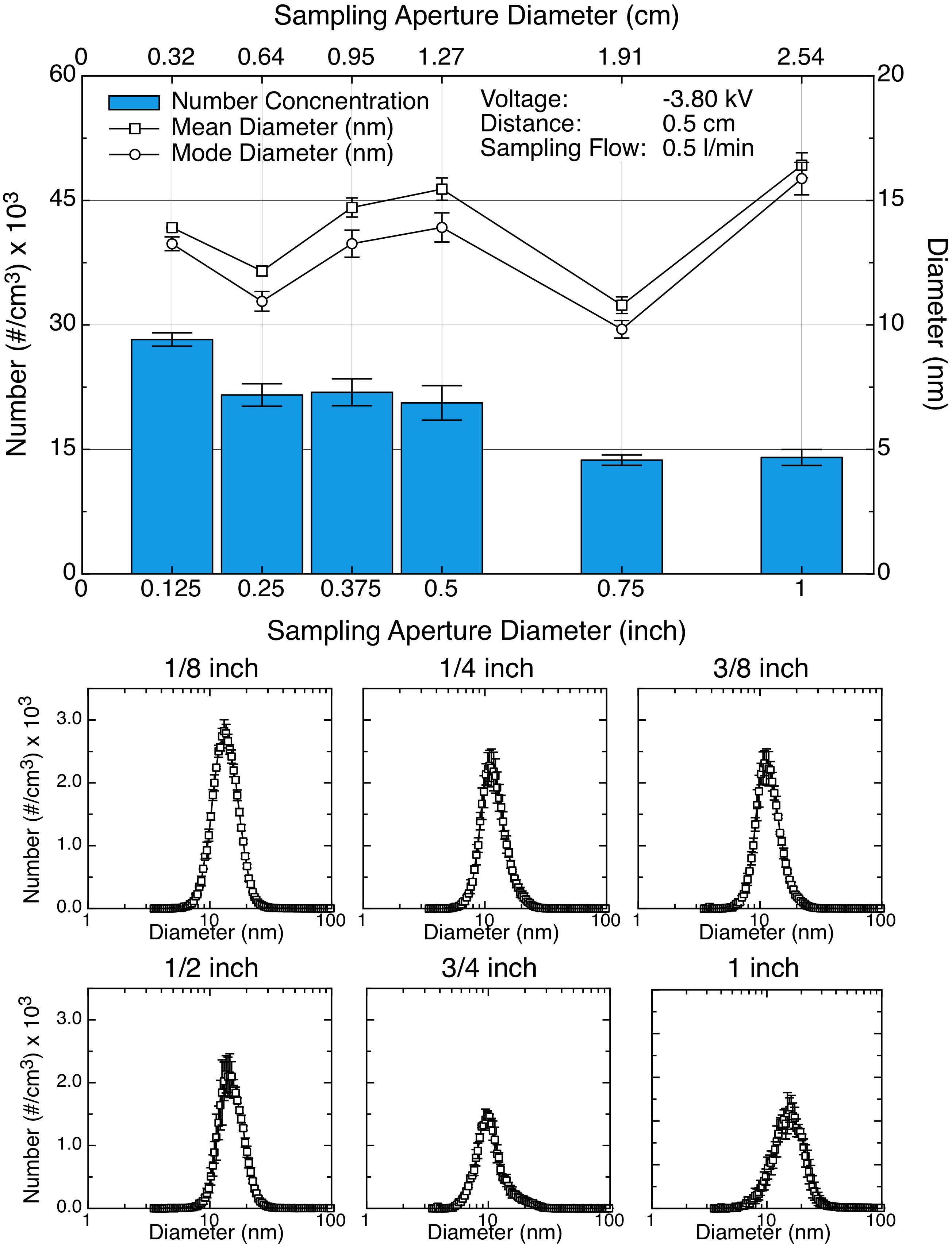


**Figure S3:** Effect of the counter electrode-sampling hole on the number of produced EWNS on the [-3.8 kV, 0.5cm] scenario. The diameter is indicative, as the SMPS is not a valid tool for the measurement.

**
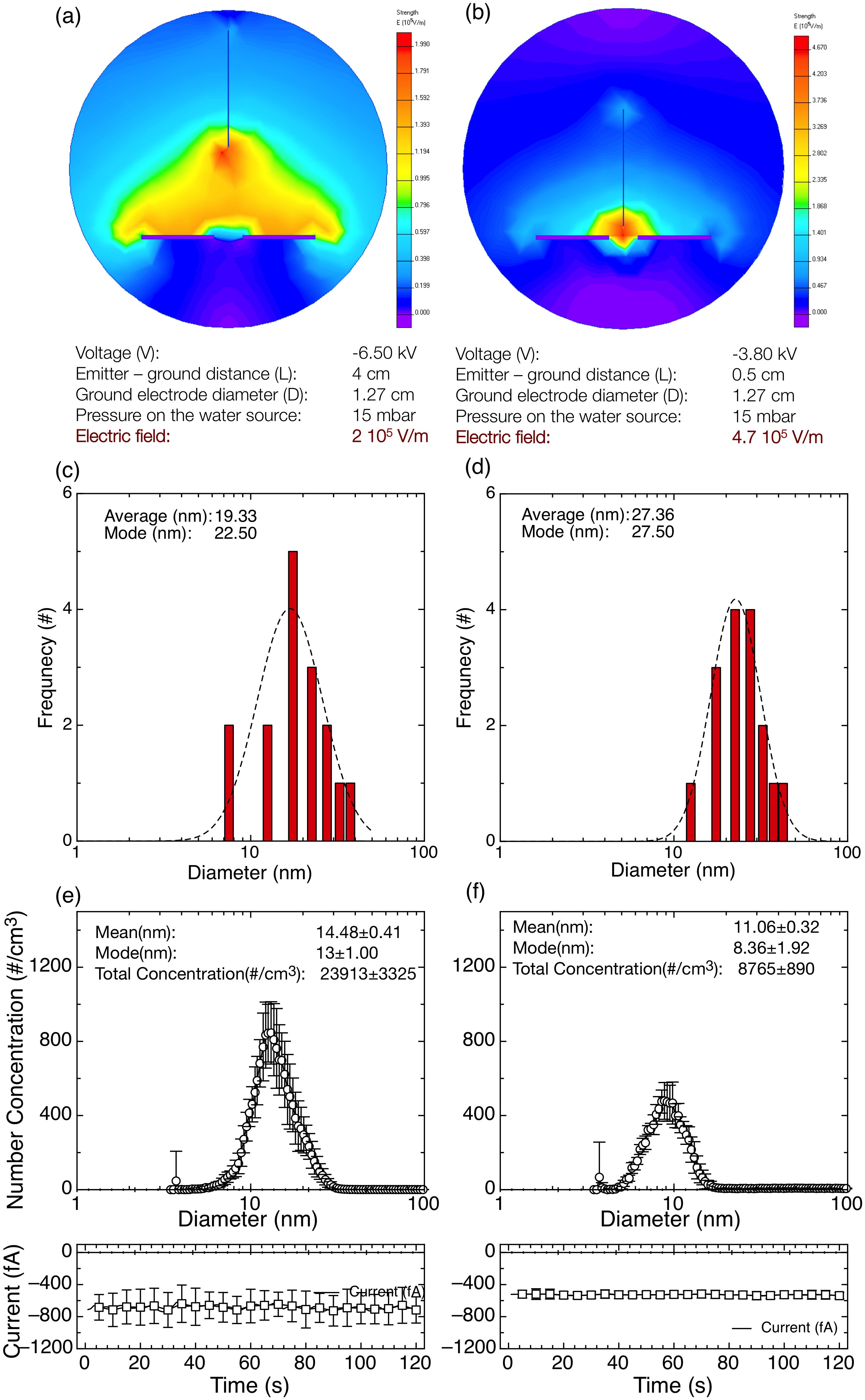
**

**Figure S4:** The simulated electric field for the two investigated scenarios (a) [6.5 kV, 4 cm] (b) [3.8 kV, 0.5 cm].


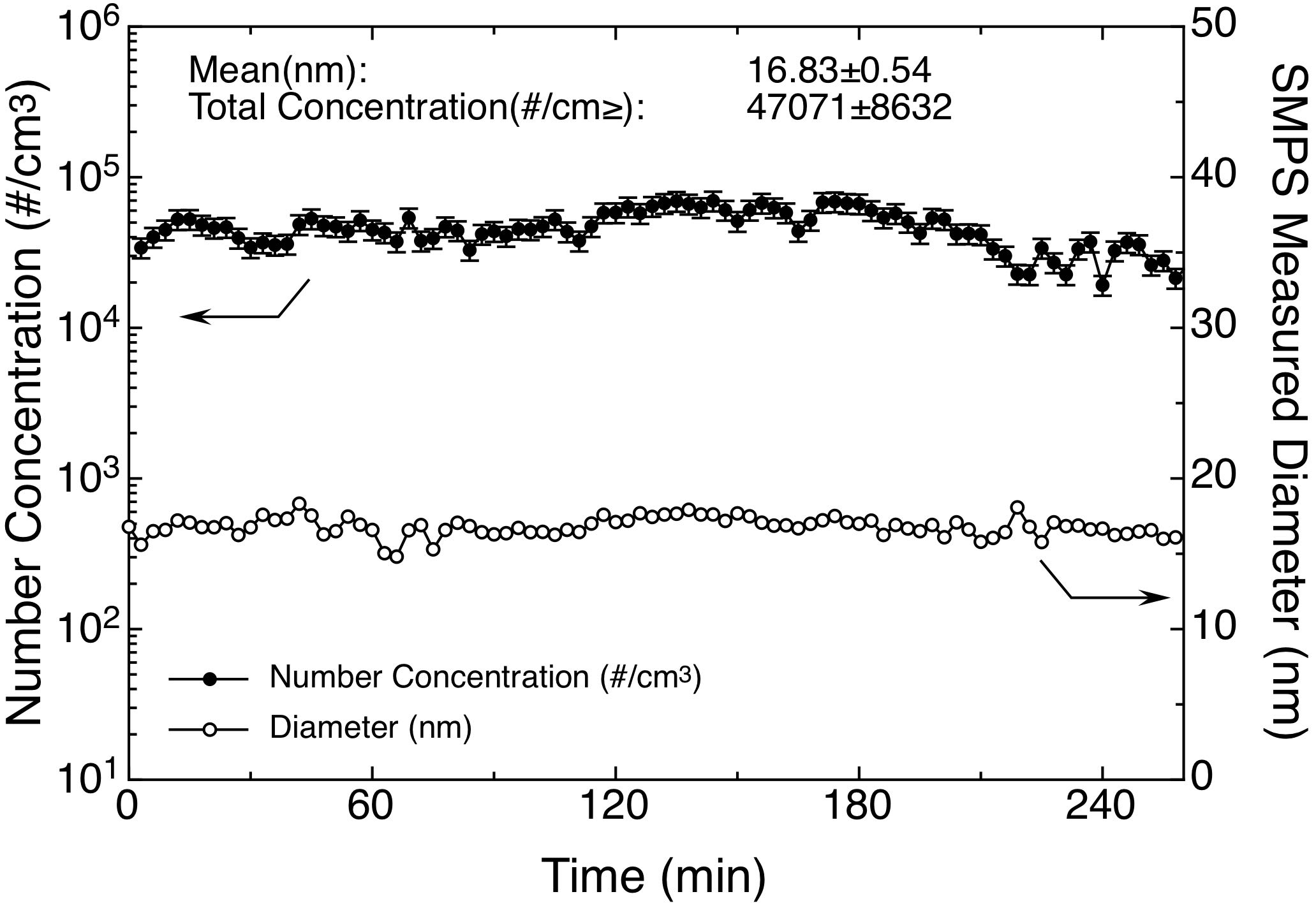


**Figure S5:** The particle number concentration as a function of time for the [-6.5kV, 4.0 cm]. The diameter is indicative of the particle size stability but not an accurate measurement.


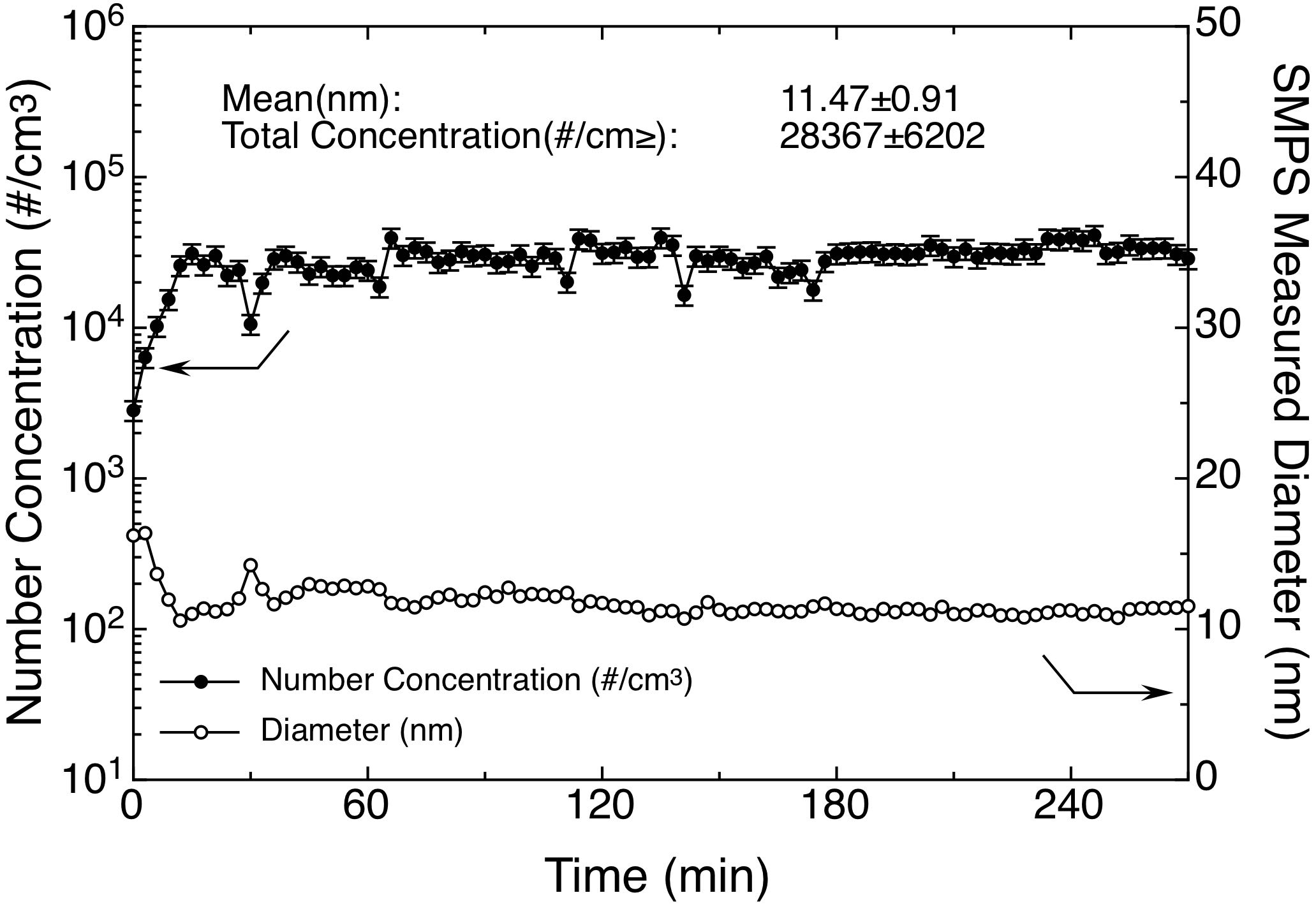


**Figure S6:** The particle number concentration as a function of time for the [-6.5kV, 4.0 cm]. The diameter is indicative of the particle size stability but not an accurate measurement.

**References:**

1. Pyrgiotakis, G. *et al.* Inactivation of Foodborne Microorganisms Using Engineered Water Nanostructures (EWNS). *Environ. Sci. Technol.* **49,** 3737–3745 (2015).
